# Functional analysis of 5 upstream polymorphic variations of the human dopamine D1 receptor gene

**DOI:** 10.1111/jcmm.14411

**Published:** 2019-05-26

**Authors:** Xue Wu, Jing‐hua Meng, Feng‐ling Xu, Bao‐jie Wang, Jun Yao

**Affiliations:** ^1^ School of Forensic Medicine China Medical University Shenyang P.R. China

## 1 BACKGROUND

We previously identified eight SNPs (single‐nucleotide polymorphisms) in the 5 regulatory region of the *DRD1* gene.^1^ However, the function of the other 5 regulatory region (−2000 bp from the transcriptional start site) and the role of the different SNP loci have not been well characterized. In this study, luciferase reporter vectors containing a single SNP locus were constructed to explore the influence of the SNPs on the 5 promoter activity of the *DRD1* gene. Moreover, the electrophoretic mobility shift assay (EMSA) and vectors containing serial deletion fragments were used to identify possible transcription factors and the DNA‐binding domain. In addition, we explored the role of three transcription factors (SP1, TFAP2B and CREB1) in the 5 promoter activity of the *DRD1* gene and expression of endogenous DRD1 in the HEK293 cell line.

## 2 METHODS

### 2.1 SNP screening and construction of luciferase‐receptor vectors

A total of nine vectors were constructed, which contained eight single SNP loci (Table S1). The vector M2 was subsequently used as the template to generate the 5 deletion vectors. The sequences of these primers are shown in Table S2.

### 2.2 Cell culture and transient transfection

Human neuroblastoma cell line SK‐N‐SH and human embryonic kidney cell line HEK293 were used to test the luciferase activity of the generated vectors.^2^

Both of the cells were seeded in 24‐well plates (1 × 10^5^ cells per well) and the pGL3 vectors (1.0 μg) were co‐transfected with Renilla luciferase (100 ng) expression vector pRL‐TK (Promega) using Lipofectamine 3000 reagent according to the manufacturer’s protocol (Invitrogen, CA, USA).

### 2.3 pGL3‐WT recombinant vector with transcription factor overexpressing vectors

Three overexpressing vectors for transcription factors CREB1, TFAP2B and SP1 (ie, pEGFP‐N1‐CREB1, pEGFP‐N1‐TFAP2B, and pEGFP‐N1‐SP1) and the pEGFP‐N1‐basic vector were purchased from Taihe Biotechnology Co. (Beijing, China).

### 2.4 Luciferase assay

Here, 24 hours after transfection, cells were harvested. Firefly luciferase activity in the cell lysates was normalized to Renilla luciferase activity. Normalized activities of all the test clones were compared to those of the clones representing the reference haplotype. Each assay was performed in triplicate in two independent experiments.

### 2.5 Electrophoretic mobility shift assay

Nuclear and cytoplasmic extracts were prepared from SK‐N‐SH and HEK293 cells using the Nuclear and Cytoplasmic Protein Extraction Kit (Beyotime, Shanghai, China) in the presence of 1% protease inhibitor (phenylmethanesulfonyl fluoride, PMSF). Here, 5‐biotinylated probes, unlabeled‐specific competitors, and nonspecific competitors were synthesized by Taihe Biotechnology Co. (Table S3).

For supershift, the antibodies were anti‐MEI1, anti‐SOX‐10, anti‐HOXA5, anti‐NR4A2 and anti‐ERRA (Santa Cruz Biotechnology, TX, USA).

### 2.6 Transcription factor‐binding prediction

The prediction software Match was used to identify putative‐binding sites for transcription factors at specific SNPs in the promoter region of the DRD1 gene (http://www.gene-regulation.com/pub/programs.html).

### 2.7 Real‐time PCR (RT‐PCR)

However, β‐actin was used as the reference gene. The mean value for the pEGFP‐N1‐basic vector control was used as a reference for statistical analysis.

### 2.8 Western blot

Total protein was extracted from HEK293 cells or HEK‐293 cells transfected with pEGFP‐N1‐CREB1, pEGFP‐N1‐TFAP2B, pEGFP‐N1‐SP1, or pEGFP‐N1‐basic using NP‐40 containing PMSF. The primary DRD1 (Thermo Scientific) and β‐actin (Abbkine, USA) antibodies were used after being diluted with TBS‐T at a ratio of 1:1000 and 1:2000, respectively.

### 2.9 Statistics

Data are presented as the mean (χ) ± SD. Differences between the two groups were determined using the analysis of variance (ANOVA) or independent samples using the *t* test. *P* < 0.05 indicated statistically significant differences. SPSS 18.0 software (SPSS, Chicago, IL, USA) was used for statistical analysis.

## 3 RESULTS

### 3.1 Luciferase assays

In HEK293 cells, there was a significant increase in luciferase activity for clones M2, M3, M6 and M7 compared to WT with M2 demonstrating the highest activity (*P* < 0.05; Figure S1). The activity of M2 increased approximately 1.4 folds as compared with the activity of the WT. There was no significant difference between the clones and WT in the SK‐N‐SH cells (Figure S2).

### 3.2 Electrophoretic mobility shift assay

**Figure 1:**
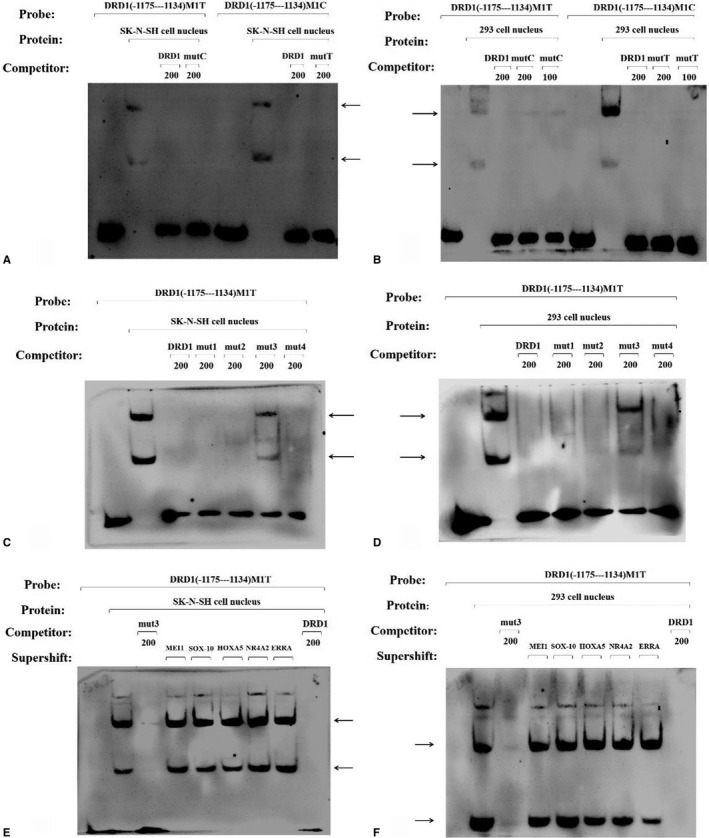
DNA‐EMSA for the T/C alleles of the rs10078866 locus using SK‐N‐SH and HEK293 nuclear extracts. The allele‐specific probes DRD1 (−1175 to 1134) M1T and DRD1 (−1175 to 1134) M1C, which span from −1175 to −1134, were used to perform the EMSA. Competitor DRD1 represents the unlabeled‐specific competing nucleotide sequences that were the same as the probe. The arrows indicate the DNA‐protein complex. (A) EMSA with SK‐N‐SH cell nuclear extracts; (B) EMSA with 293 cell nuclear extracts; (C) DNA‐protein complex in the mut3 competitor in SK‐N‐SH cell nuclear extract; (D) DNA‐protein complex in the mut3 competitor in 293 cell nuclear extract; (E) No supershift bands were observed in SK‐N‐SH cell nuclear extract; (F) No supershift bands were observed in 293 cell nuclear extract

We conducted EMSA using specific probes to detect whether the rs10078866 locus could affect DNA‐protein interactions because the M2 vector showed the highest luciferase activity in HEK293 cells. As shown in Figure 1A‐D, EMSA with SK‐N‐SH and HEK293 nuclear extracts revealed that two specific complexes were formed by the T and C alleles of the rs10078866 locus, respectively. However, the complexes could not be abolished by the unlabeled‐specific competitors, which indicated that this DNA‐protein binding was not specific for the T/C alleles. To ascertain the exact binding sequence in the DNA‐protein complexes, four mutated oligonucleotides were used as non‐specific competitors (Table S2). We observed the DNA‐protein complex in the mut3 competitor in both the SK‐N‐SH and HEK293 nuclear extract (Figure 1C and 1). The results indicated that the mutated sequence of the mut3 competitor (ACTTTGAGC, from −1153 to −1145 of the *DRD1* gene) was the binding sequence of the complex. Prediction of the transcription factor binding‐site alteration caused by the rs10078866 locus in the ACTTTGAGC oligonucleotide was performed (Figure S3). Based on the prediction results, a supershift experiment was conducted using specific transcription factor antibodies (Figure 1E and 1). However, no supershift bands were observed with the anti‐MEI1, anti‐SOX‐10, anti‐HOXA5, anti‐NR4A2 and anti‐ERRA antibodies.

### 3.3 Luciferase assays of pGL3‐WT co‐transfected with the transcription factor overexpressing vectors

In HEK293 cells, the luciferase activity increased significantly after pEGFP‐N1‐TFAP2B transfection, approximately 0.5 times as the pEGFP‐N1‐basic transfection, but decreased after pEGFP‐N1‐CREB1 or pEGFP‐N1‐SP1 transfection (Figure S4). In SK‐N‐SH cells, the luciferase activity increased significantly following transfection with pEGFP‐N1‐CREB1 and pEGFP‐N1‐SP1, but not pEGFP‐N1‐TFAP2B (Figure S5).

### 3.4 The role of three transcription factors in endogenous expression of DRD1

The mRNA expression of endogenous DRD1 decreased slightly 48 hours after pEGFP‐N1‐CREB1 transfection into HEK293 cells but increased significantly after pEGFP‐N1‐TFAP2B or pEGFP‐N1‐SP1 were transfected (Figure S6A). The mRNA expression levels gradually returned to basal levels between 48 and 96 hours after the transfection (Figure S6B and C).

**Figure 2:**
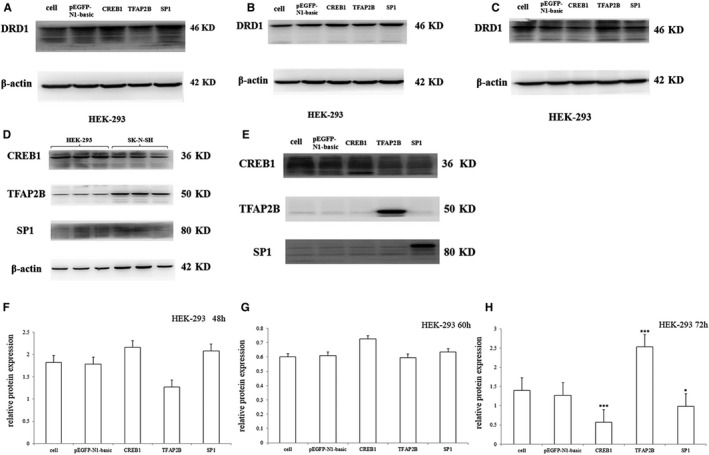
Endogenous DRD1 protein expression following the transcription factor overexpression in HEK293 cells. A‐C, 48, 60 and 72 h after transfection, respectively; (D) the endogenous expression of the transcription factors in HEK293 and SK‐N‐SH cell lines; (E) the overexpression of the transcription factors following transfection of HEK293 cells; (F‐H) the normalized DRD1 protein expression 48, 60 and 72 h after transfection. Normalized DRD1 protein levels following CREB1, TFAP2B and SP1 overexpressing were compared to the reference vector (pEGFP‐N1‐basic). The error bars represent the standard deviation of the mean. *P < 0.05; ***P < 0.001

The endogenous expression of transcription factors (CREB1, TFAP2B, and SP1) was detected in both HEK‐293 and SK‐N‐SH cells (Figure 2D).

The exogenous expression of the three transcription factors was detectable at 48 hours after transfection of HEK293 cells (Figure 2E). There were no obvious changes in the endogenous DRD1 expression levels from 48 to 60 hours after transfection (Figure 2A, 2, 2, and G). However, 72 hours after transfection, both CREB1 and SP1 downregulated, and TFAP2B upregulated endogenous DRD1 expression (Figure 2C and H). For TFAP2B, it increased the expression of the endogenous DRD1 about one‐fold as compared with the negative control (pEGFP‐N1‐basic), while the expression of the endogenous DRD1 was reduced by 50% under the effect of CREB1.

## 4 DISCUSSION

In this study, the C allele of the rs10078866 locus significantly increased luciferase activity in HEK293 cells, but not in SK‐N‐SH cells. This heterogeneity might be due to the differences in cell type.^2^, ^3^, ^4^, ^5^ EMSA demonstrated that there was no influence of the T/C alleles on the formation of DNA‐protein complexes, and ACTTTGAGC (from −1153 to −1145 of *DRD1* gene) could form a DNA‐protein complex in the presence of SK‐N‐SH and HEK293 nuclear extracts. In addition, the endogenous expression of DRD1 in HEK293 cells could be regulated by the CREB1, TFAP2B and SP1 transcription factors. Additional experiments are needed to better understand the function of the 5 regulatory region of the *DRD1* gene in future.

## CONFLICT OF INTEREST

None.

## AUTHOR CONTRIBUTIONS

Jun Yao conceived and designed the experiments. Xue Wu and Feng‐ling Xu performed the experiments. Feng‐ling Xu, Jing‐hua Meng and Bao‐jie Wang analysed the data. Jun Yao wrote the paper.
