# Supplementary material for: Functional analysis of 5 upstream polymorphic variations of the human dopamine D1 receptor gene
Source: J Cell Mol Med. 2019 May 26;23(8):5813–7. doi: 10.1111/jcmm.14411 (PMC6653764; doi:10.1111/jcmm.14411)

Table S1. The polymorphism distribution of nine constructed vectors containing eight SNPs.

| SNP | rs267410 | rs10078866 | rs10063995 | rs10078714 | rs201568565 | rs146439723 | rs35916350 | rs199763328 |
| --- | --- | --- | --- | --- | --- | --- | --- | --- |
| WT | T | T | C | T | C | C | G | G |
| M1 | G | T | C | T | C | C | G | G |
| M2 | T | C | C | T | C | C | G | G |
| M3 | T | T | A | T | C | C | G | G |
| M4 | T | T | C | C | C | C | G | G |
| M5 | T | T | C | T | T | C | G | G |
| M6 | T | T | C | T | C | T | G | G |
| M7 | T | T | C | T | C | C | C | G |
| M8 | T | T | C | T | C | C | G | T |

Note: WT vector was designed as the reference vector. Underlined text indicates the mutated base of the SNP locus.

Table S2. Primers used for cloning of two 5’ deletion fragments.

| Primer name |  | Sequence |
| --- | --- | --- |
| *Kpn*I-*DRD1*(-1159)F |  | 5'-GGGGTACCCCTGTGACTTTGAGCAGGCC-3' |
| *Kpn*I-*DRD1*(-1134)F |  | 5'-GGGGTACCCCCTTTCCGAGCCGCAT-3' |
| *Bgl*II-*DRD1*(+20)R |  | 5'-GAAGATCTTCGCAATGCGAGGCAGCGAG-3' |

Note: Underlined text indicates the introduction of a restriction site for *Kpn*I or *Bgl*II; F, forward; R, reverse.

Table S3.Primers used for EMSA.

| Primer name |  | Sequence |
| --- | --- | --- |
| Probes |  |  |
| DRD1(-1175---1134)M1T |  | 5'-CGGCTCCCGCGTGAGCTGTG**T**GACTTTGAGCAGGCCCCACTAC-3' |
| DRD1(-1175---1134)M1C |  | 5'-CGGCTCCCGCGTGAGCTGTG**C**GACTTTGAGCAGGCCCCACTAC-3' |
| Competitors |  |  |
| DRD1(-1175---1134)mutT |  | 5'-CGGCTCCCGCGTGAGCTGTG**T**GACTTTGAGCAGGCCCCACTAC-3' |
| DRD1(-1175---1134)mutC |  | 5'-CGGCTCCCGCGTGAGCTGTG**C**GACTTTGAGCAGGCCCCACTAC-3' |
| DRD1(-1175---1134)mut1 |  | 5'-CTTACAAATAAGGAGCTGTGTGACTTTGAGCAGGCCCCACTAC-3' |
| DRD1(-1175---1134)mut2 |  | 5'-CGGCTCCCGCGTTGATCACTGAACTTTGAGCAGGCCCCACTAC-3' |
| DRD1(-1175---1134)mut3 |  | 5'-CGGCTCCCGCGTGAGCTGTGTGCAGGCTCATAGGCCCCACTAC-3' |
| DRD1(-1175---1134)mut4 |  | 5'-CGGCTCCCGCGTGAGCTGTGTGACTTTGAGCCTATTATGTTAC-3' |

Note: Bold text indicates the specific base corresponding to the allele of the rs10078866 locus. Underlined text represents the mutated sequence corresponding to the probe DRD1(-1175---1134)M1T.

**Figure legends**

Fig. S1. Relative luciferase activity of nine vectors in HEK293 cells.

The normalized activities of all the test clones (M1 to M8) were compared to the reference vector (WT). * represents *p* < 0.05. The error bars represent the standard deviation of the mean.


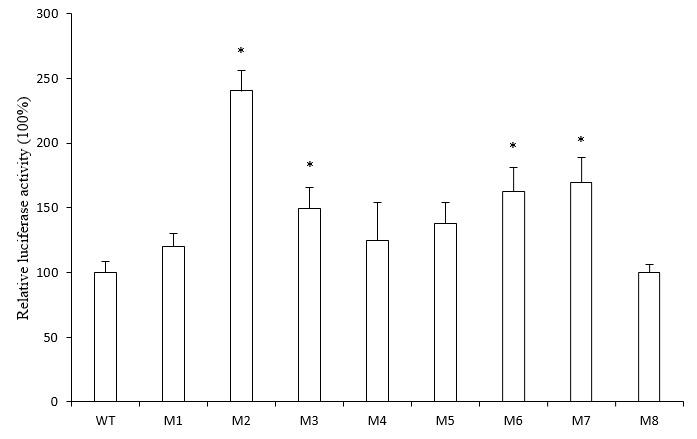


Fig. S2. Relative luciferase activity of nine vectors in SK-N-SH cells.

Normalized activities of all the test clones (M1 to M8) were compared to the reference vector (WT). * represents *p* < 0.05. The error bars represent the standard deviation of the mean.


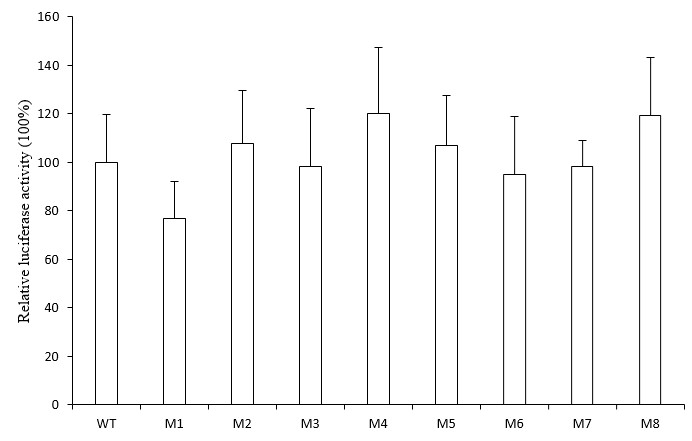


Fig. S3. Prediction of transcription factor binding-site alterations caused by the rs10078866 locus in the human *DRD1* gene 5’ regulatory region.


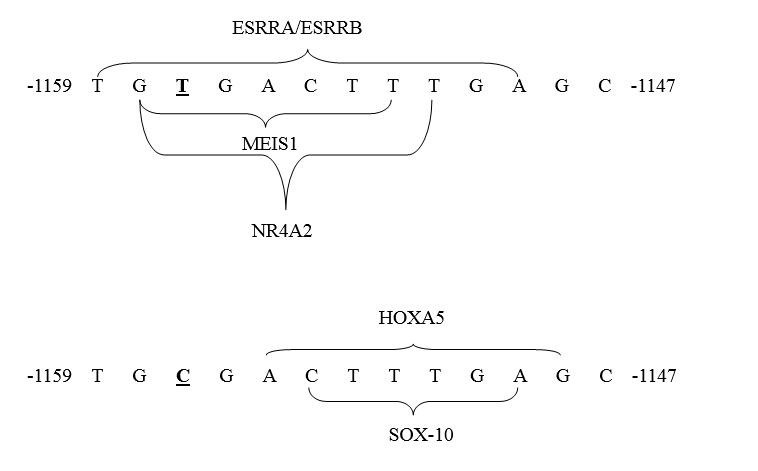


Fig. S4. Relative luciferase activity of pGL3-WT in the presence of transcription factor overexpressing vectors in HEK293 cells.

Normalized activities of WT+CREB1, WT+TFAP2B, and WT+SP1 were compared to the reference vector (WT+basic). The error bars represent the standard deviation of the mean. *, *p* < 0.05; **, *p* < 0.01; ***, *p* < 0.001


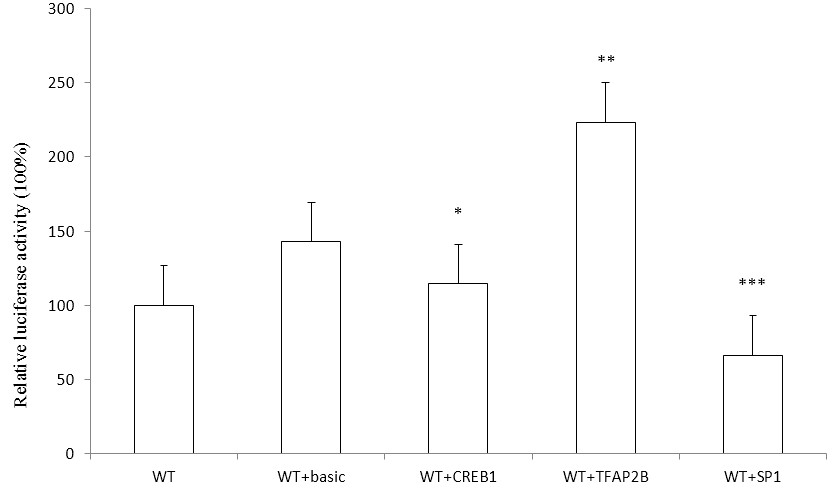


Fig. S5. Relative luciferase activity of pGL3-WT in the presence of transcription factor overexpressing vectors in SK-N-SH cells.

Normalized activities of WT+CREB1, WT+TFAP2B, and WT+SP1 were compared to the reference vector (WT+basic). The error bars represent the standard deviation of the mean. *, *p* < 0.05; **, *p* < 0.01; ***, represents *p* < 0.001.


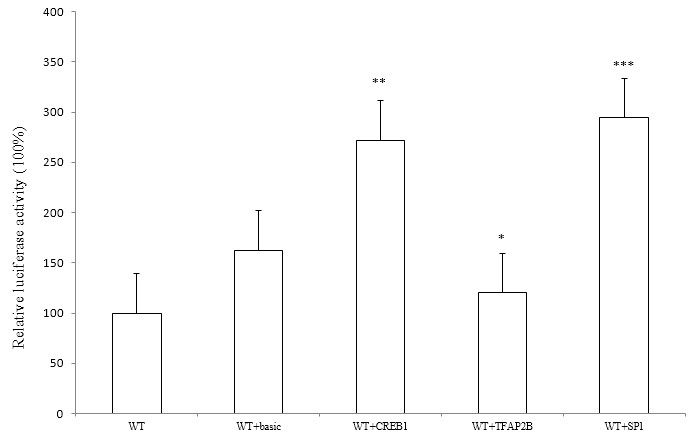


Fig. S6. Endogenous DRD1 mRNA expression following transcription factor overexpression in HEK293 cells.

Normalized DRD1 mRNA levels following CREB1, TFAP2B, or SP1 overexpression were compared to the reference vector (basic). The error bars represent the standard deviation of the mean. *, *p* < 0.05; **, *p* < 0.01; ***, *p* < 0.001

Note: a, b, and c represent 48 h, 72 h, and 96 h after transfection, respectively.


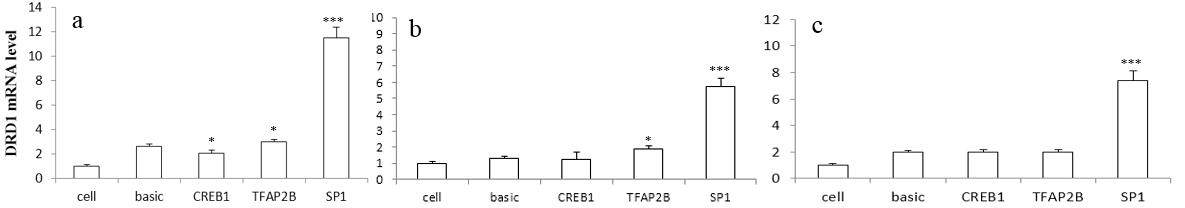

Supplement: Supplementary file 1 [file JCMM-23-5813-s001.doc]
